# Correction for Sharma et al., “Specific and Randomly Derived Immunoactive Peptide Mimotopes of Mycobacterial Antigens”

**DOI:** 10.1128/msphere.00609-24

**Published:** 2024-09-05

**Authors:** Archna Sharma, Abhik Saha, Surajit Bhattacharjee, Subrata Majumdar, Sujoy K. Das Gupta

## AUTHOR CORRECTION

Vol. 13, no. 10, p. 1143–1154, 2006, https://doi.org/10.1128/cvi.00127-06. In Fig. 2, the right half of lane 5 appears similar to the corresponding region in lane 7. This similarity is possibly an artifact, and the results do not affect the main conclusions of the paper. Lane 1, representing the molecular marker, was cut and pasted. Unfortunately, we do not have access to the raw data to provide a corrected figure.

In Fig. 7A and B, the images were part of the same experiment but included inadvertent duplication of the preimmune lanes. Additionally, there are inadvertent duplications in the immune lanes in panel A, PepCS-35[12] lanes R2 and R3, and panel B, PepCS-35[7] lanes R2 and R3. We regret these errors. We do not have access to the raw data and cannot provide a correction. However, these results were supportive information, and in the subsequent experiment, in Fig. 7C, both positive and negative controls were incorporated. Thus, the conclusions remain unchanged.

